# Common and Specific Functional Activity Features in Schizophrenia, Major Depressive Disorder, and Bipolar Disorder

**DOI:** 10.3389/fpsyt.2019.00052

**Published:** 2019-02-19

**Authors:** Yongfeng Yang, Shu Liu, Xiaoyan Jiang, Hongyan Yu, Shuang Ding, Yanli Lu, Wenqiang Li, Hongxing Zhang, Bing Liu, Yue Cui, Lingzhong Fan, Tianzi Jiang, Luxian Lv

**Affiliations:** ^1^Key Laboratory for Neuroinformation of Ministry of Education, School of Life Science and Technology, University of Electronic Science and Technology of China, Chengdu, China; ^2^Department of Psychiatry, Henan Mental Hospital, The Second Affiliated Hospital of Xinxiang Medical University, Xinxiang, China; ^3^Henan Key Laboratory of Biological Psychiatry, Xinxiang Medical University, Xinxiang, China; ^4^Brainnetome Center, Institute of Automation, Chinese Academy of Sciences, Beijing, China; ^5^National Laboratory of Pattern Recognition, Institute of Automation, Chinese Academy of Sciences, Beijing, China; ^6^University of Chinese Academy of Sciences, Beijing, China; ^7^The Queensland Brain Institute, University of Queensland, Brisbane, QLD, Australia

**Keywords:** schizophrenia, major depressive disorder, bipolar disorder, magnetic resonance imaging, functional activity

## Abstract

**Objectives:** Schizophrenia (SZ), major depressive disorder (MDD), and bipolar disorder (BD) are serious mental disorders with distinct diagnostic criteria. They share common clinical and biological features. However, there are still only few studies on the common and specific brain imaging changes associated with the three mental disorders. Therefore, the aim of this study was to identify the common and specific functional activity and connectivity changes in SZ, MDD, and BD.

**Methods:** A total of 271 individuals underwent functional magnetic resonance imaging: SZ (*n* = 64), MDD (*n* = 73), BD (*n* = 41), and healthy controls (*n* = 93). The symptoms of SZ patients were evaluated by the Positive and Negative Syndrome Scale. The Beck Depression Inventory (BDI), and Beck Anxiety Inventory (BAI) were used to evaluate the symptoms of MDD patients. The BDI, BAI, and Young Mania Rating Scale were used to evaluate the symptoms of MDD and BD patients. In addition, we compared the fALFF and functional connectivity between the three mental disorders and healthy controls using two sample *t*-tests.

**Results:** Significantly decreased functional activity was found in the right superior frontal gyrus, middle cingulate gyrus, left middle frontal gyrus, and decreased functional connectivity (FC) of the insula was found in SZ, MDD, and BD. Specific fALFF changes, mainly in the ventral lateral pre-frontal cortex, striatum, and thalamus were found for SZ, in the left motor cortex and parietal lobe for MDD, and the dorsal lateral pre-frontal cortex, orbitofrontal cortex, and posterior cingulate cortex in BD.

**Conclusion:** Our findings of common abnormalities in SZ, MDD, and BD provide evidence that salience network abnormality may play a critical role in the pathogenesis of these three mental disorders. Meanwhile, our findings also indicate that specific alterations in SZ, MDD, and BD provide neuroimaging evidence for the differential diagnosis of the three mental disorders.

## Introduction

Schizophrenia (SZ), major depressive disorder (MDD), and bipolar disorder (BD) are three common but serious mental disorders with unknown causes. According to the World Health Organization, the burden of mental disorders will account for >1/4 of the total burden of disease by 2020 (1). SZ, MDD, and BD have a genetic basis: twin, adoption and family-based prospective studies have confirmed that all three are highly hereditary (2–4). The genetic variations shared between SZ and BD, BD and MDD, SZ and MDD are 15, 10, and 9%, respectively (5). SZ, BD, and MDD have similar endophenotype characteristics (6, 7), and neuropsychological mechanisms (8, 9). At present, the diagnosis of mental disorder is typically through the symptom criteria specified by the Diagnostic and Statistical Manual of Mental Disorders (DSM)-5 or 10th revision of the International Classification of Diseases, and there is a lack of effective and objective biological markers.

The study of resting state functional magnetic resonance imaging (fMRI) has been widely used to explore the biological markers of mental disorders (10–12). Measurement of the amplitude of low frequency fluctuations (ALFF) (13, 14) and functional connectivity (FC) are the main research methods in this field. The current fMRI studies mostly focus on the common abnormalities of ALFF or FC between SZ and BD, or SZ and MDD, or MDD and BD. For instance, SZ and BD have FC abnormalities in the default mode network (DMN) [including the pre-frontal cortex (PFC) and medial PFC], and frontal occipital network (15–17). Furthermore, SZ and MDD have been found to have widespread DMN (18) and central executive network (CEN) disruption (19, 20), and the decreased FC in amygdala-PFC network may be a potential marker for these mental disorders (21). Both MDD and BD also have significantly increased ALFF in the left ventral anterior cingulate cortex (ACC) (22) and decreased short-range FC strength in the bilateral precuneus (23). Meanwhile, specific FC abnormalities in SZ (16, 21), BD (15, 17), and MDD (21, 23) have also found been found in fMRI studies.

As far as we know, most previous studies have only compared SZ and BD, or SZ and MDD, or MDD and BD. However, some studies have suggested that the three mental disorders may have common and specific structural and functional imaging features (24, 25). However, fMRI studies on the common and specific features of SZ, MDD, and BD are scare and inconsistent in findings. Based on the above research, we hypothesized that SZ, MDD, and BD may have common specific features identifiable through brain imaging. Therefore, this study explored the common and specific abnormalities in SZ, MDD, and BD. We predicted that imaging data would support the differential diagnosis of SZ, MDD, and BD.

## Materials and Methods

### Participants and Clinical Diagnosis

The study protocol was in accordance with principles of the Declaration of Helsinki and approved by the Ethics Committee of the Second Affiliated Hospital of Xinxiang Medical University (Xinxiang, China). Written informed consent was obtained from all participants after the study aims and procedures were fully explained, and participants were aware that they could withdraw from the study at any time. None of the authors had access to information that could identify individual participants during or after data collection.

There were four groups of participants: SZ (*n* = 64), BD (*n* = 41), MDD (*n* = 73), and healthy controls (HCs; *n* = 93). Data were collected at the Second Affiliated Hospital of Xinxiang Medical University (Xinxiang, China) between March 2013 and October 2017. All participants were right handed. HCs had no history of psychiatric or neurological disease. Participants were screened by psychiatrists using simple non-structured interviews and met the diagnostic criteria of DSM-IV for SZ, BD, or MDD. Exclusion criteria were: other mental disorders; organic causes of depression including heart, liver, or kidney disease; presence of surgically implanted electronic devices or metal frames preventing fMRI scanning.

Psychiatric symptoms of SZ patients were evaluated using the Positive and Negative Syndrome Scale (PANSS) (26). The Beck Anxiety Inventory (BAI) (27), and Beck Depression Inventory (BDI) (28) were used to evaluate the symptoms of MDD patients. The BDI, BAI, and Young Mania Rating Scale (YMRS) (29) were used to evaluate the symptoms of BD patients.

### Data Acquisition and Preprocessing

fMRI was carried out on a 3.0-T MR system equipped with an MR header coil (Siemens, Verio, Germany). Whole-brain magnetization transfer images were acquired using a three-dimensional, fast, low-angle shot sequence. fMRI: slices = 50, repetition time (TR)/echo time (TE) = 2,000/30 ms, field of view (FOV) = 220 × 220 mm^2^, matrix = 64 × 64, flip angle = 90°, voxel size of mm = 3.4 × 3.4 × 4.0, slice thickness = 4 mm. T1: slices = 50, TR/TE = 2,530/2.43 ms, FOV = 256 × 256 mm^2^, matrix = 256 × 256, flip angle = 70°, voxel size of mm = 1.0 × 1.0 × 1.0, slice thickness = 1 mm. T2: slices = 192, TR/TE = 4,000/96 ms, FOV = 256 × 256 mm^2^, matrix = 256 × 256, flip angle = 150°, voxel size of mm = 0.7 × 0.7 × 5.0, slice thickness = 5 mm.

The same preprocessing procedures were applied to each dataset, and BRANT (Brainnetome fMRI Toolkit, https://github.com/kbxu/brant) was used to preprocess the images. The whole preprocessing procedures consisted of: (1) slice timing; (2) head motion correction; (3) spatial normalization; (4) temporal band-pass filtration (0.01–0.08 Hz); (5) smoothing with a 6 mm Gaussian kernel; and (6) removing the noise by multiple regression of linear trends, mean signal of global brain, white matter (WM) and cerebrospinal fluid (CSF) and Friston's 24 parameter head motion model. No participant had a head motion of >2 mm or a spin of >2° in any direction throughout the resting-state scans.

### Fractional ALFF (fALFF) and FC Calculation

fALFF calculation was performed using BRANT. ALFF is a reliable method for describing spontaneous brain activity, but there are some specific cisternal areas which show significantly higher ALFF because of physiological noise (30, 31). The fALFF method was proposed to selectively suppress artifacts from non-specific brain areas. This method can significantly improve the sensitivity and specificity of spontaneous brain activity detection (31), and was therefore used in this study. Specifically, the fast Fourier transform was first used to transform the time course of each voxel signal into the corresponding power spectrum in the frequency domain. Then, we took the square root at each frequency component of the power spectrum to obtain the amplitude of this frequency component of the original time series in the time domain. Finally, the average amplitude at each voxel was obtained across 0.01–0.1 Hz, providing fALFF maps (32).

According to the fALFF analysis for the SZ, MDD, BD, and HC datasets, the region that had the same change across the three mental disorders compared with HCs was chosen as the region of interest (ROI). FC between the ROI and whole brain was then calculated using BRANT for each participant. Specifically, the Pearson's correlation coefficients between the average BOLD time series in the ROI and the time series from all the voxels were first computed, and then the resulting correlations were then transformed to approximate a Gaussian distribution using Fisher's *z* transformation, providing the FC map for each subject.

### Meta-Analysis of the Three Mental Disorders

The meta-analysis results for SZ, MDD, and BD were obtained from Neurosynth (http://neurosynth.org/analyses/terms), which is a platform for large-scale, automated synthesis of fMRI data. It automatically identified all studies in the database that loaded highly on the terms SZ, MDD, and BD, and then performed meta-analyses to identify the brain regions that were consistently or preferentially reported in the results of those studies (33). We chose the forward statistical inference map. *Z*-scores corresponded to the likelihood that a region would activate if a study used a particular term. The obtained maps were corrected with a false discovery rate approach, with an expected false discovery rate of 0.01. The automated meta-analysis contained 577 studies associated with SZ, 343 studies associated with MDD or depressive disorders, and 111 studies associated with BD.

### Statistical Analyses

We used SPM12 (http://www.fil.ion.ucl.ac.uk/spm/) to perform two sample *t*-tests for fALFF between the three mental disorders and HCs. Age and sex were included in the model as covariates. The Alphasim algorithm was performed with a multiple comparison correction using REST (34). The estimated smooth kernel was 6 mm and statistical maps were thresholded at a voxel-level of *P* < 0.001 and cluster size >12 or 13 to reach a cluster-level significance of alpha < 0.05. The ROI was then selected from the overlap between statistical maps. For FC between the ROI and whole brain in the four datasets, the analysis of two sample *t*-tests and the same multiple comparison correction was conducted again to identify the common variation in FC. Statistical maps were also thresholded at a voxel-level of *P* < 0.001 and cluster size >12 or 13 to reach a cluster-level significance of alpha < 0.05.

## Results

### Demographic Characteristics and Clinical Information

The demographic and clinical attributes of the four groups are shown in Table 1. There were no significant differences in gender or age between three mental disorders (SZ, MDD, and BP) and HC.

**Table 1:** Demographic characteristics of the participants.

**Variables**	**SZ (*n* = 64)**	**MDD (*n* = 73)**	**BD (*n* = 41)**	**HC (*n* = 93)**
Gender(male/female)	28/36	27/46	23/18	46/47^a^
Age (years old)	28.55 ± 4.41	32.61 ± 9.27	32.98 ± 9.41	30.29 ± 6.78^b^
Education (years)	11.05 ± 2.75	11.17 ± 3.81	10.49 ± 3.79	11.43 ± 2.98
Age of Onset (years)	22.58 ± 6.05	31.97 ± 11.27	26.98 ± 9.82	N/A
Duration of Illness (months)	37.41 ± 38.90	37.10 ± 55.08	80.41 ± 74.37	N/A
Episode (first/recurrent)	23/41	37/36	7/34	N/A
Medication (yes/no)	60/4	59/14	41/0	N/A
Antipsychotics (yes/no)	60/4	5/68	25/16	N/A
Antidepressants (yes/no)	0/64	62/11	16/25	N/A
Mood stabilizer (yes/no)	0/64	3/70	25/16	N/A
PANSS	80.67 ± 8.85	N/A	N/A	N/A
BDI	N/A	21.91 ± 7.13	9.95 ± 11.58	N/A
BAI	N/A	32.58 ± 14.95	30.34 ± 12.69	N/A
YMRS	N/A	N/A	24.35 ± 10.67	N/A

*SZ, Schizophrenia; MDD, Major Depressive Disorder; BD, Bipolar Disorder; HC, Healthy Control; PANSS, Positive and Negative Syndrome Scale; BDI, Beck Depression Inventory; BAI, BecK Anxiety Inventory; YMRS, Young Mania Rating Scale*.

a *P-values which represent the case-control difference in gender: HC vs. SZ = 0.482, HC vs. MDD = 0.063, HC vs. BD = 0.483*.

b *P-values which represent the case-control difference in age: HC vs. SZ = 0.074, HC vs. MDD = 0.060, HC vs. BD = 0.064*.

*N/A, not applicable*.

### Functional Activity Analysis

**Figure 1:**
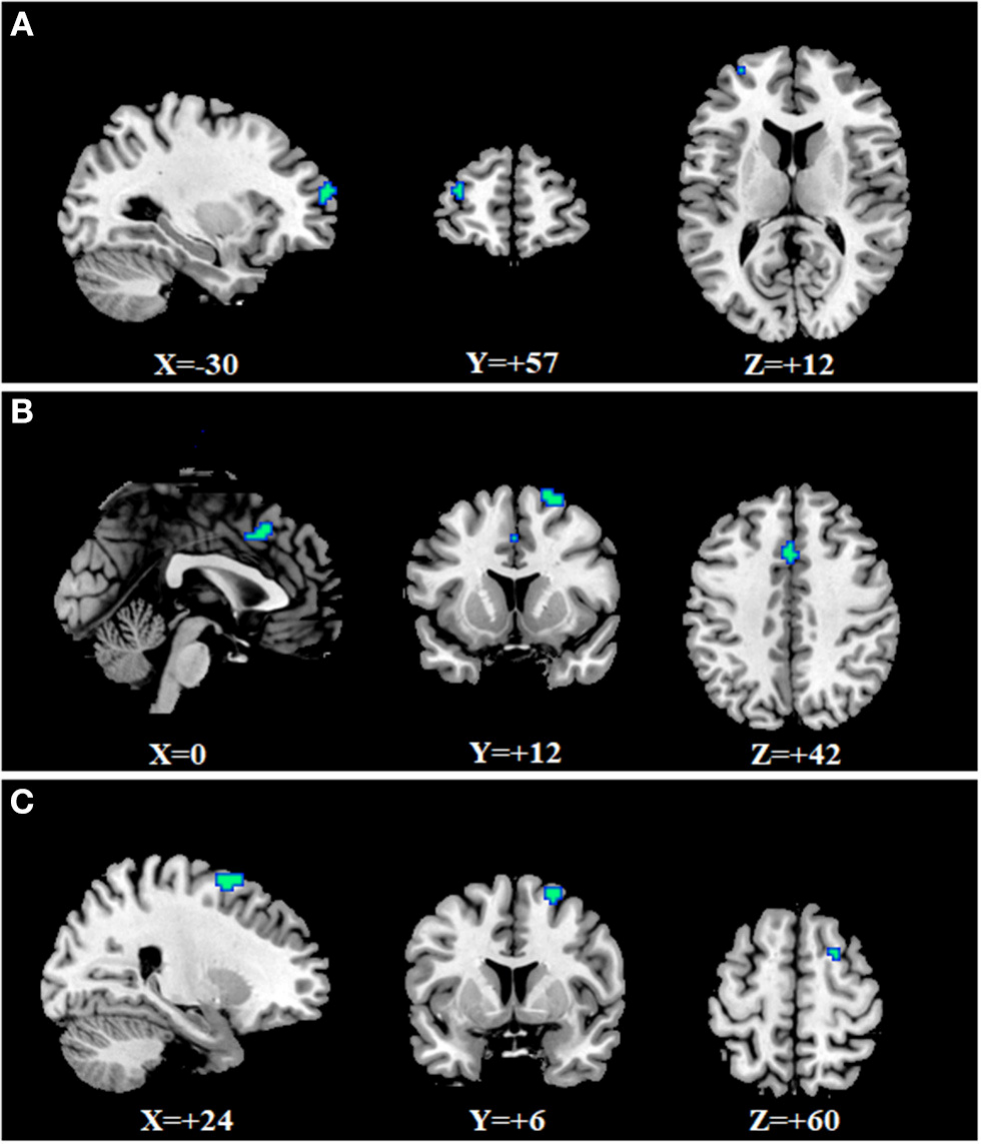
Common changes of fALFF in SZ, MDD, and BD. **(A)** right superior frontal gyrus (−30, 57, 12); **(B)** middle cingulate gyrus (0, 12, 42); **(C)** left middle frontal gyrus (24, 6, 0).

To identify whether there were differences for fALFF between the three mental disorders and HCs, two sample *t*-tests were conducted (Supplementary Figure 1). By overlapping the changes in the three mental disorders, we found three regions that changed simultaneously (Figure 1 and Supplementary Table 1). The first region was in the right superior frontal gyrus (SFG) which corresponds to the right SFG in the automated anatomical labeling (AAL) atlas (peak Montreal Neurological Institute (MNI) coordinate: −30, 57, 12). The values of fALFF all decreased in SZ, MDD, and BD groups, compared with HCs (Figure 1A). The second region was in the middle cingulate gyrus, which is included in the left and right middle cingulate gyrus in the AAL atlas (peak MNI coordinate: 0, 12, 42). Similarity, the values of fALFF also dropped in all three mental disorders (Figure 1B). The third region was in left middle frontal gyrus (MFG). It is also a part of the left MFG in the AAL atlas (peak MNI coordinate: 24, 6, 60). In this region, the values of fALFF in the three mental disorders were smaller than those in HCs (Figure 1C). Being aware of the effects and limitations of global signal regression (GSR), we further conducted an analysis of fALFF without GSR, and the results are shown in the Supplementary Materials (Supplementary Figure 2). The case-control differences in fALFF without GSR were significantly smaller than those with GSR. No significant association was found between the values of fALFF in three common regions and PANSS, BDI, BAI, and YMRS (*P* > 0.05).

**Figure 2:**
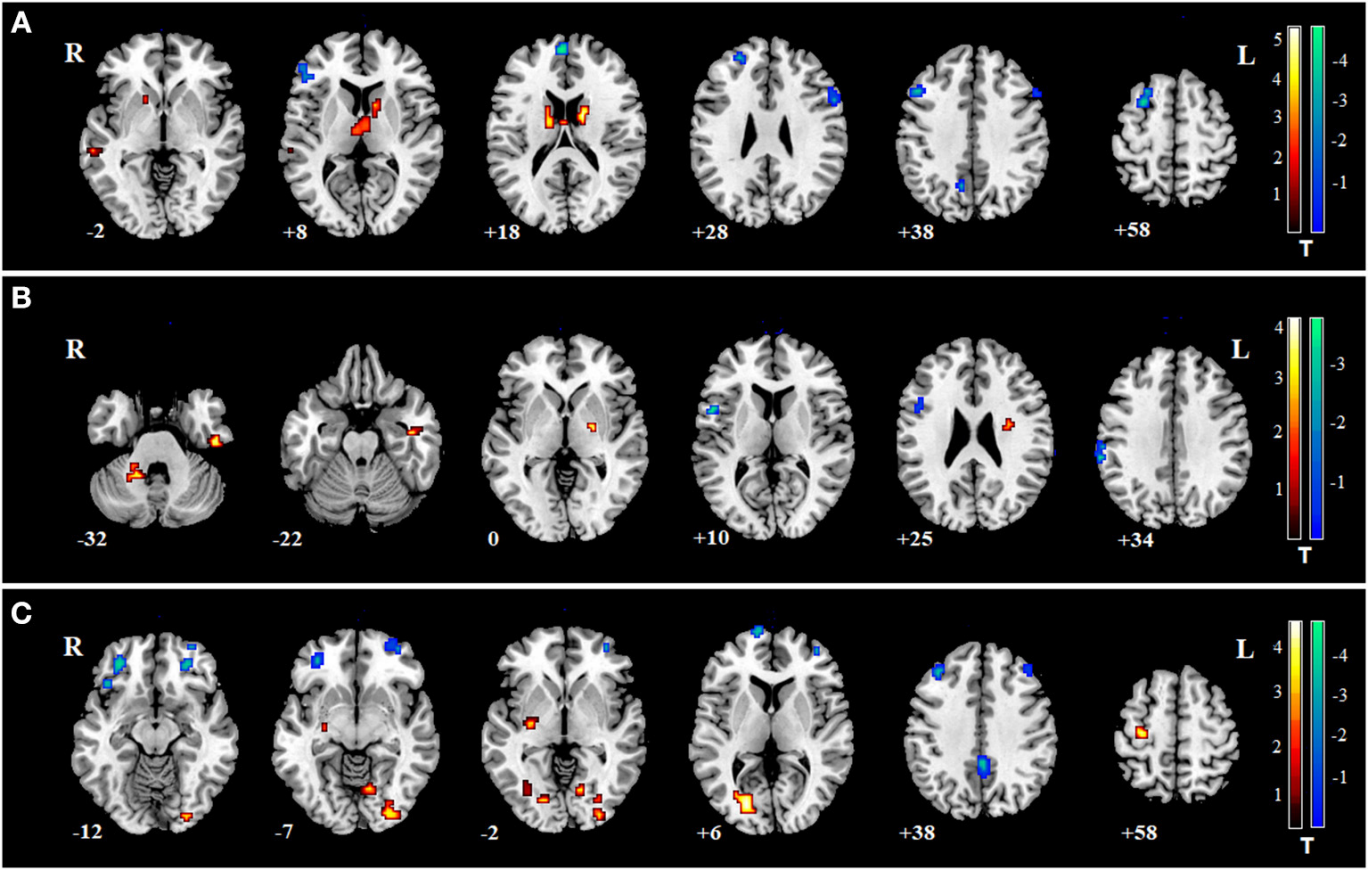
Specific changes of fALFF in SZ, MDD, and BD. Warm colors indicate that the values of fALFF in disorders were larger than in HCs; Cool colors represent the values of fALFF in disorders which were smaller than in HCs. **(A)** for SZ, fALFF was increased in the left middle temporal gyrus, bilateral caudate, and thalamus, and decreased in some frontal regions such as bilateral triangularis of the inferior frontal gyrus, left medial superior frontal gyrus, and left middle frontal gyrus. **(B)** for MDD, fALFF was increased in the cerebellum anterior lobe and right inferior temporal gyrus, and decreased in the left pre-central gyrus and left supramarginal gyrus. **(C)** for BD, fALFF was increased in the right inferior occipital gyrus, right lingual gyrus, left putamen, left middle occipital gyrus, and left pre-central gyrus, and decreased in bilateral orbital middle frontal gyrus, left superior frontal gyrus, bilateral posterior cingulum and bilateral middle frontal gyrus.

We found specific changes of fALFF in some regions in the three mental disorders (Supplementary Table 1 and Figures 2A–C). Specific fALFF changes were found mainly in the ventral lateral pre-frontal cortex, striatum and thalamus for SZ, left motor cortex and parietal lobe for MDD, dorsal lateral pre-frontal cortex, orbitofrontal cortex, and posterior cingulate cortex for BD.

### Functional Connectivity in the Whole Brain

**Figure 3:**
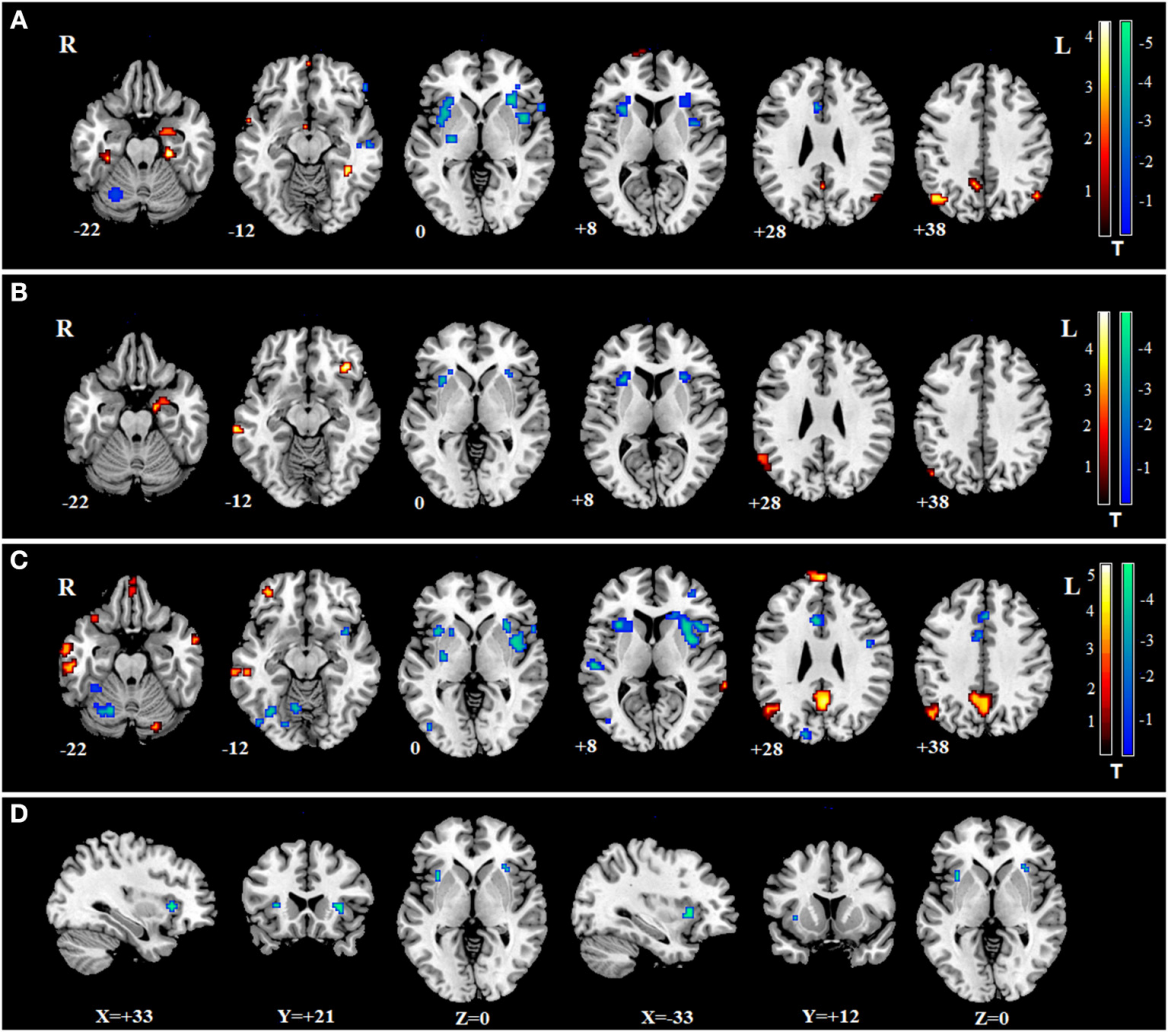
The changes of FC between the ROI and whole brain in SZ, MDD, and BD. Warm colors indicate the values of FC in disorders were larger than in HCs; Cool colors represent the values of FC in disorders which were smaller than in HCs. **(A)** changes of FC in SZ; **(B)** changes of FC in MDD; **(C)** changes of FC in BD; **(D)** two regions of the common changes of FC in the three mental disorders: left insula (−33, 12, 0) and right insula (33, 21, 0).

The decreased FCs of the cingulate gyrus in the patients of SZ, MDD, and BD all have been reported in previous studies, compared with controls (35–37). Therefore, we hypothesized the FCs between the cingulate gyrus and some specific regions may represent common biomarkers for the three mental disorders. From the three regions with common changes of fALFF in the three mental disorders, the region in the middle cingulate gyrus was selected as the ROI. FC between ROI and whole brain was calculated in SZ, MDD, BD, and HCs. Two sample *t*-tests were conducted between three patient groups and HC group (Figures 3A–C). When overlapping the changes in the three mental disorders, only FC between the ROI and right insula and left insula were found (Figure 3D). No significant association was found between the FC of the insula and PANSS, BDI, BAI, or YMRS in the three mental disorders (*P* > 0.05).

**Figure 4:**
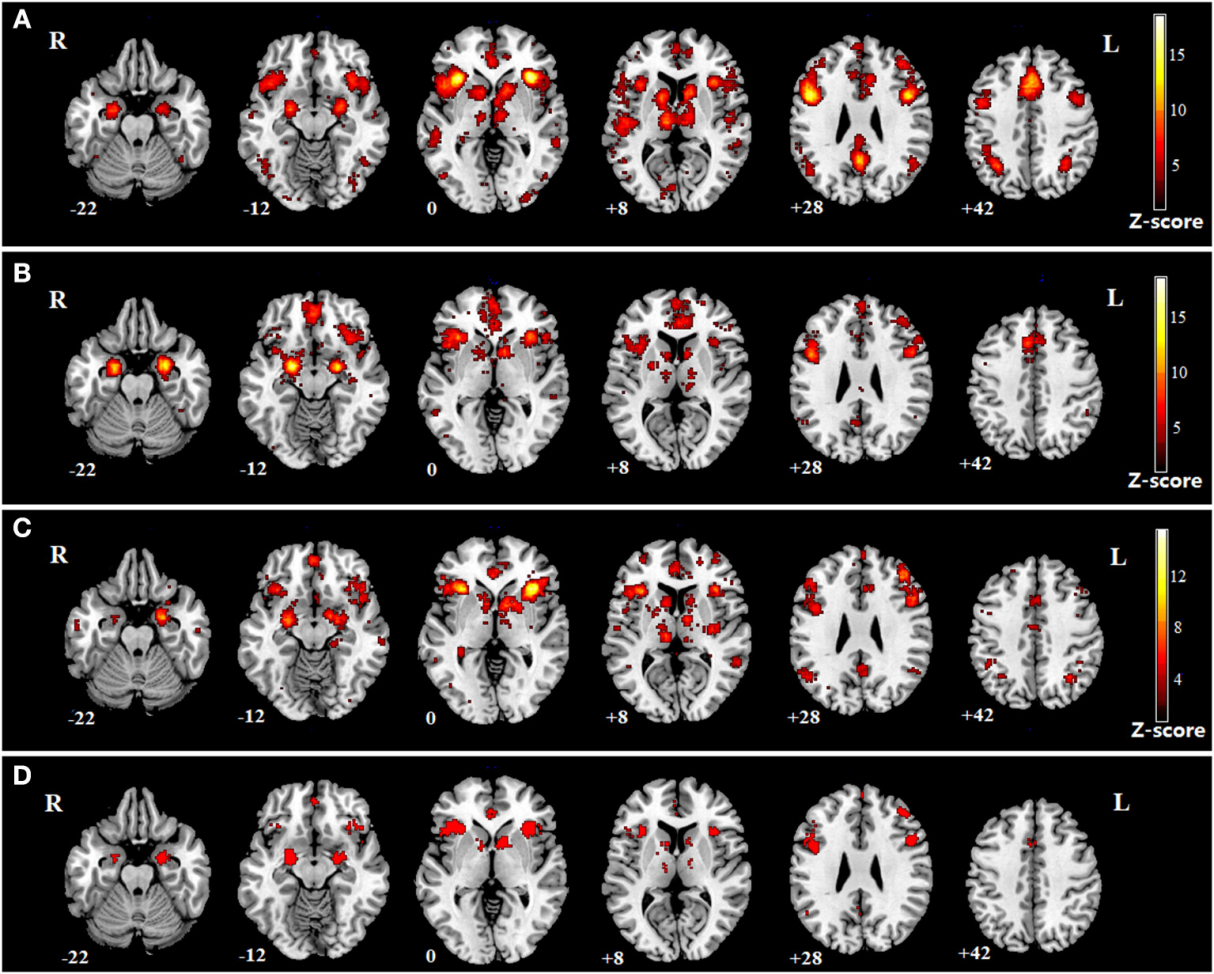
Meta-analysis of SZ, MDD, and BD. **(A)** impaired regions in SZ; **(B)** impaired regions in MDD; **(C)** impaired regions in BD; **(D)** common impaired regions in the three mental disorders.

The impaired regions in SZ, MDD, and BD according to the meta-analysis of previous studies are shown (Figures 4A–C). The impaired regions in all three mental disorders are also shown, which include both the left and right insula (Figure 4D).

## Discussion

In this study, we found significantly decreased functional activity in the right SFG, middle cingulate gyrus, and left MFG in SZ, MDD and BD. The decreased FCs of the cingulate gyrus in the patients with SZ, MDD, and BD have been reported in previous studies (35–37). Therefore, we hypothesized that the FCs between the cingulate gyrus and some specific regions may be common biomarkers for the three mental disorders and chose the middle cingulate gyrus as the ROI. We also found common decreased FC in the insula for the three mental disorders. Meanwhile, specific changes associated with each of the three mental disorders were also found. This study further confirmed our previous hypothesis and provides evidence for the significant common and specific functional activity features in SZ, MDD, and BD.

Previous studies have reported common FC abnormalities in the PFC for SZ and BD (15, 17), between the amygdaloid and PFC for SZ and MDD (21) and between the left ventral anterior cingulate and left amygdala for MDD and BD (22). Meanwhile, specific abnormalities in FC associated with SZ have been found in the amygdala-dorsal ACC (16, 21), hippocampus, fusiform gyrus (17), medial PFC, ACC, posterior cingulate cortex (PCC), precuneus (15), mesolimbic pathway, and frontotemporal lobe (16). Specific increased ALFF have been found in the right brain regions and bilateral cerebellum, and decreased ALFF in the bilateral calcarine fissure and the bilateral lingual gyrus in BD (22, 23). MDD has specific abnormalities in FC in the amygdala-ventral PFC (21), medial orbitofrontal cortex (OFC) (38), and bilateral cerebellum (23). Recently, a structural MRI study in a Chinese population reported the gray matter volumes of paralimbic and heteromodal cortices and the white matter integrity of the callosal, limbic-paralimbic-heteromodal, cortico-cortical, thalamocortical, and cerebellar regions, and found significant differences between the SZ, BD, MDD, and HC groups (24). This study further supports the observation of common brain imaging changes in SZ, BD, and MDD at the structural imaging level through exploring the relative similarities and the differences.

Our fMRI study explored the similarities and the differences in SZ, BD, and MDD. A novel finding was that three mental disorders had significantly decreased functional activity in the right SFG, middle cingulate gyrus, left MFG, and decreased FC in the insula. Moreover, the abnormality in the insula was consistent with our meta-analysis of previous studies. The SFG and MFG are located in the PFC of the human brain (39). Previous fMRI studies have indicated that the SFG is involved in self-awareness and coordination with the sensory system (40). Meanwhile, the PFC brain region has been implicated in planning complex cognitive behavior, personality expression, decision making, and moderating social behavior (41). The PFC area is also associated with executive function and emotional regulation (42). The cingulate gyrus, an integral part of the limbic system, is involved with emotion formation and processing (43), as well as executive function, and is highly important in SZ (44) and depression (45). The insula, through the thalamus, receives information and sends output information to limbic-related structures (46), and plays an important role in emotional control (47, 48). Moreover, SZ, MDD, and BD have similar clinical features relating to emotional instability (6). Thus, our findings provide evidence for the SFG, MFG, middle cingulate gyrus, and insula all having a role in emotional regulation. A recent study also found common decreased short-range connectivity patterns across SZ, MDD, and BD (25). Therefore, our studies along with that of Xia et al., provide further evidence for common FC abnormality in SZ, MDD, and BD.

The GSR was performed in the preprocessing of fMRI data. We found inconsistent results relating to the case-control differences in fALFF between analyses with and without GSR. However, whether the preprocessing should include GSR is controversial (49–51). Researchers are left in a dilemma regarding whether to use GSR or not because of the lack of an accepted gold standard (52). GSR can improve the specificity of positive correlations and the correspondence to anatomical connectivity (53). It can also help remove non-neuronal sources of global variance such as respiration (54) and movement (55). Therefore, GSR is widely used as a preprocessing technique for fMRI analysis. However, GSR may remove BOLD signal fluctuations of neuronal origin (56), spuriously weakening some correlations. It has also been reported to drive artifactual group differences in FC (57, 58). The case-control differences in fALFF without GSR were significantly smaller than those with GSR, a finding in line with a previous fMRI study that also reported that GSR had an impact on the final results (59). However, previous studies reported that their main findings with and without GSR remained consistent (60, 61). In sum, it is hard to determine whether or not regress out the global signal in the preprocessing of fMRI data.

The salience network (SN) comprises the bilateral insula and ACC and plays a role in recruiting relevant brain regions for the processing of sensory information (35, 62). Previous studies have indicated dysfunctional SN activity in SZ and MDD (35, 63). Meanwhile, DMN (64) and CEN (65) abnormalities have been identified in SZ and MDD (63). Furthermore, the SN modulates the interaction between the internally oriented DMN and the externally oriented CEN (66, 67). Therefore, SN characteristics have gradually become a crucial factor in identifying dysfunction in psychopathology (63). Our results suggested a common abnormality in FC in the insula across in SZ, MDD, and BD. Therefore, another important finding was the abnormality of SN which may play a role in the pathogenesis of these there mental disorders.

Findings indicated specific fALFF changes, mainly in the ventral lateral pre-frontal cortex, striatum, and thalamus for SZ, the left motor cortex and parietal lobe for MDD, and the dorsal lateral pre-frontal cortex, orbitofrontal cortex, and posterior cingulate cortex for BD. Previous studies have found that FC is decreased for amygdala-dACC (21) and PFC (38) in Chinese SZ patients. Interestingly, our findings mostly focus on the ventral lateral PFC, and this is also consistent with previous research in a Chinese population (38). Meda et al. found that connectivity is decreased in the PCC and precuneus in BD (15), and our findings are consistent with this. Our study found decreased fALFF in the right lingual gyrus in BD. This result is partially consistent with a previous study that found significantly decreased ALFF in the bilateral lingual gyrus in BD (22). BD had significantly increased ALFF in the right SFG, hippocampus, amygdala, inferior temporal gyrus, OFC (22), mesolimbic pathway and frontotemporal lobe (16), and significantly decreased ALFF in the bilateral calcarine fissure (22), hippocampus, fusiform gyrus (17), medial PFC, and ACC (15). Although our results are not consistent with those studies, our research provides new evidence for specific fALFF changes in BD. MDD was associated with decreased FC in the amygdala-ventral PFC (21) and the bilateral cerebellum (23). Our study found significant fALFF increases in the cerebellum anterior lobe in MDD. An explanation for why our study is inconsistent with previous results may be due to 62.4% of the all patients being in a recurrent episode in our study, compared to 72.9% (24) and 67.8% (25) of the all patients undergoing their first episode in previous research. However, our study provides a major finding relating to patients undergoing recurrent episodes. These findings enrich the theories of functional activity in SZ, MDD, and BD.

There are several limitations in this study. First, due to difficulties during data collection, the number of patients with BD was relatively insufficient, and the next stage for this research strategy is to increase the sample size with multi-center studies. Second, all participants with SZ, MDD, or BD were taking psychotropic medication, and the study was underpowered to consider the potential effects of these medications. However, previous studies have reported no significant differences between medicated and non-medicated patients in SZ, MDD, and BD (24). However, further studies need to verify these results in medication-naïve samples. Finally, at present, our study and previous research (24, 25) into the three mental disorders have mainly concentrated on the Chinese Han population, therefore, further research is needed to explore multiple countries and multiple ethnic groups.

## Conclusion

In summary, we found significant common abnormality of functional activity and FC in the SZ, MDD, and BD. Our findings provide evidence for the SFG, MFG, middle cingulate gyrus, and insula all having a role in emotional regulation in these mental disorders. Meanwhile, those results implicate that dysfunction of the SN may play a role in the pathogenesis of the three mental disorders. Furthermore, our findings also indicate that specific alterations in SZ, MDD, and BD provide neuroimaging evidence for the differential diagnosis of the three mental disorders. Further studies should explore the applicability and veracity of these findings in the diagnosis and differential diagnosis of the three mental disorders.

## Author Contributions

LL and TJ designed the study and wrote the protocol. YY, SL, and XJ managed the literature searches and analyses. YY, HY, SD, YL, WL, and HZ conducted sample selection and data management. YY, SL, YC, BL, and LF undertook the statistical analysis, and YY, SL, XJ, and BL wrote the first draft of the manuscript. All authors contributed to and have approved the final manuscript.

### Conflict of Interest Statement

The authors declare that the research was conducted in the absence of any commercial or financial relationships that could be construed as a potential conflict of interest.

## Supplementary Material

Supplementary Figure 1 The changes of fALFF in SZ, MDD, and BD. Warm colors indicate that the values of fALFF in disorders are larger than those in HC; Cool colors represent that the values of fALFF in disorders are smaller than those in HC. **(A)** The changes of fALFF in SZ; **(B)** The changes of fALFF in MDD; **(C)** The changes of fALFF in BD.

Supplementary Figure 2 The changes of fALFF without GSR in SZ, MDD, and BD. Warm colors indicate that the values of fALFF in disorders are larger than those in HC; Cool colors represent that the values of fALFF in disorders are smaller than those in HC. **(A)** The changes of fALFF in SZ; **(B)** The changes of fALFF in MDD; **(C)** The changes of fALFF in BD.

Supplementary Table 1 The common and specific changes in SZ, MDD and BD.
