# Supplementary figures and images for: Common and Specific Functional Activity Features in Schizophrenia, Major Depressive Disorder, and Bipolar Disorder
Source: Front Psychiatry. 2019 Feb 19;10:52. doi: 10.3389/fpsyt.2019.00052 (PMC6389674; doi:10.3389/fpsyt.2019.00052)

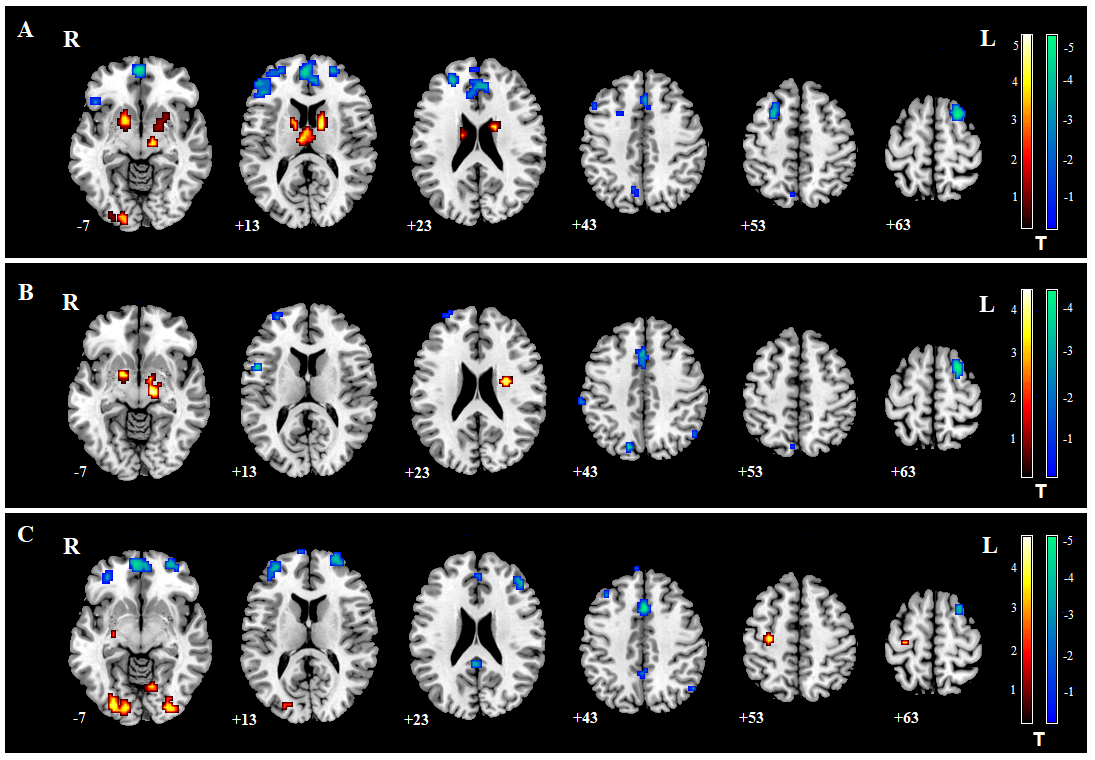

Supplement: Supplementary Figure 1 — The changes of fALFF in SZ, MDD, and BD. Warm colors indicate that the values of fALFF in disorders are larger than those in HC; Cool colors represent that the values of fALFF in disorders are smaller than those in HC. (A) The changes of fALFF in SZ; (B) The changes of fALFF in MDD; (C) The changes of fALFF in BD. [file Image_1.TIF]

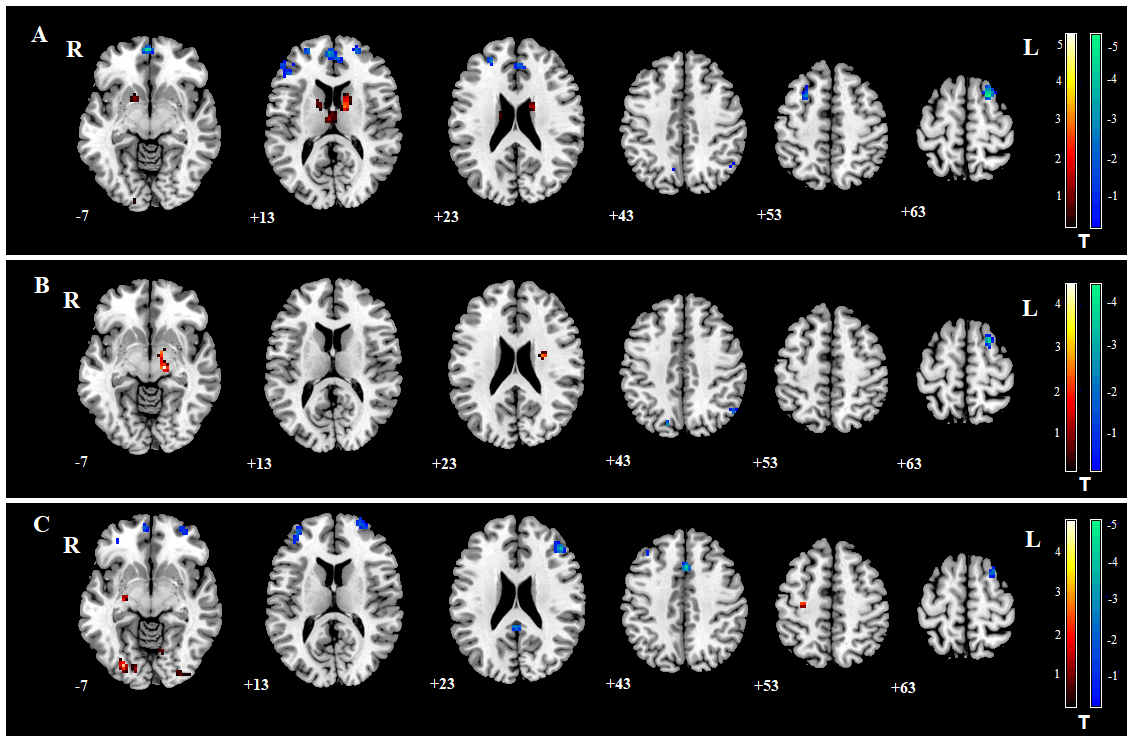

Supplement: Supplementary Figure 2 — The changes of fALFF without GSR in SZ, MDD, and BD. Warm colors indicate that the values of fALFF in disorders are larger than those in HC; Cool colors represent that the values of fALFF in disorders are smaller than those in HC. (A) The changes of fALFF in SZ; (B) The changes of fALFF in MDD; (C) The changes of fALFF in BD. [file Image_2.TIF]
