# Supplementary material for: Common and Specific Functional Activity Features in Schizophrenia, Major Depressive Disorder, and Bipolar Disorder
Source: Front Psychiatry. 2019 Feb 19;10:52. doi: 10.3389/fpsyt.2019.00052 (PMC6389674; doi:10.3389/fpsyt.2019.00052)
Supplement: Supplementary Table 1 — The common and specific changes in SZ, MDD and BD. [file Table_1.pdf]

Supplementary Table 1. The common and specific changes in SZ, MDD and BD.

	Region(AAL)	Disorders	Size	Coordinate	Peak intensity
Common changes	Frontal_Mid_L (-30, 57, 12)	SZ	32	(-24, 45, 24)	-4.7361
		MDD	15	(-30, 60, 18)	-4.0800
		BD	58	(-33, 54, 9)	-4.8019
	Cingulum_Mid_L/R (0, 12, 42)	SZ	286	(0, 57, -6)	-5.3572
		MDD	20	(0, 21, 45)	-3.8620
		BD	87	(3, 18, 42)	-4.8780
	Frontal_Sup_R (24, 6, 60)	SZ	47	(24, 12, 63)	-5.6098
		MDD	41	(24, 9,63)	-4.5162
		BD	13	(24, 12, 66)	-3.9896
Specific changes	Temporal_Mid_L	SZ	13	(-60, -30, 0)	3.9076
	Caudate_R	SZ	45	(15, 0, 18)	5.3861
	Thalamus_L/R	SZ	48	(-6, -15, 12)	4.4814
	Frontal_Inf_Tri_L	SZ	27	(-42, 33, 12)	-4.0564
	Caudate_L	SZ	26	(-15, -6, 21)	4.2429
	Frontal_Sup_Medial_L	SZ	15	(-3, 57, 18)	-4.6343
	Frontal_Mid_L	SZ	12	(-24, 48, 24)	-4.3571
	Frontal_Inf_Tri_R	SZ	21	(54, 18, 23)	-4.3991
	Frontal_Mid_L	SZ	12	(-45, 21, 39)	-3.7807
	Frontal_Mid_L	SZ	45	(-24, 12, 51)	-4.6374
	Cerebellum Anterior Lobe	MDD	28	(-21, -42, -39)	3.9188
	Temporal_Inf_R	MDD	33	(48, -18, -30)	3.8744
	Precentral_L	MDD	12	(-51, 9, 12)	-3.9262
	SupraMarginal_L	MDD	18	(-63, -33, 36)	-3.5863
	Frontal_Mid_Orb_R	BD	31	(30, 42, -15)	-3.9361
	Frontal_Mid_Orb_L	BD	41	(-33, 42, -9)	-4.2824
	Occipital_Inf_R	BD	40	(30, -90, -3)	3.7883
	Frontal_Mid_Orb_R	BD	24	(30, 57, -9)	-4.1352
	Lingual_R	BD	18	(15, -69, -3)	4.0456
	Putamen_L	BD	14	(-27, -15, -3)	3.6223

Occipital_Mid_L	BD	73	(-27, -78, 6)	4.9027
Frontal_Sup_L	BD	13	(-12, 72, 9)	-3.7135
Cingulum_Post_L/R	BD	87	(3, -45, 30)	-4.8780
Frontal_Mid_R	BD	59	(42, 36, 30)	-4.7228
Frontal_Mid_L	BD	17	(-36, 33, 39)	-3.9802
Precentral_L	BD	28	(-30, -18, 57)	4.6312

Abbreviations : AAL, automated anatomical labeling; SZ, schizophrenia; MDD, major depressive disorder; BD, bipolar disorder.
